# Physical and mental health among caregivers: findings from a cross-sectional study of Open University students in Thailand

**DOI:** 10.1186/1471-2458-12-1111

**Published:** 2012-12-26

**Authors:** Vasoontara Yiengprugsawan, David Harley, Sam-ang Seubsman, Adrian C Sleigh

**Affiliations:** 1 National Centre for Epidemiology and Population Health, The Australian National University, Building 62, Mills Rd, Acton 2601, Canberra, ACT, Australia; 2 School of Human Ecology, Sukhothai Thammathirat Open University, Nonthaburi, Thailand

**Keywords:** Carer, Caregiver, Self-assessed health, Psychological distress, Lower back pain, Thai cohort study

## Abstract

**Background:**

Caregivers constitute an important informal workforce, often undervalued, facing challenges to maintain their caring role, health and wellbeing. Little is known about caregivers in middle-income countries like Thailand. This study investigates the physical and mental health of Thai adult caregivers.

**Methods:**

This report derives from distance-learning students working and residing throughout Thailand and recruited for a health-risk transition study in 2005 (N=87,134) from Sukhothai Thammathirat Open University. The cohort follow-up questionnaire in 2009 (N = 60,569) includes questions on caregiver status which were not available in 2005; accordingly, this study is confined to analysis of the 2009 data. We report cross-sectional associations between caregiver status and health.

**Results:**

Among the study participants in 2009, 27.5% reported being part-time caregivers and 6.6% reported being full-time caregivers. Compared to male non-caregivers, being a part-time or full-time male caregiver was associated with lower back pain (covariate-Adjusted Odds Ratios, AOR 1.36 and 1.67), with poor psychological health (AOR 1.16 and 1.68), but not with poor self-assessed health. Compared to female non-caregivers, being a part- or full-time female caregiver was associated with lower back pain (AOR 1.47 and 1.84), psychological distress (AOR 1.32 and 1.52), and poor self-assessed health (AOR 1.21 and 1.34).

**Conclusions:**

Adult caregivers in Thailand experienced a consistent adverse physical and mental health burden. A dose–response effect was evident, with odds ratios higher for full-time caregivers than for part-time, and non-caregivers. Our findings should raise awareness of caregivers, their unmet needs, and support required in Thailand and other similar middle-income countries.

## Background

As populations age in many parts of the world, caregivers are becoming increasingly numerous, yet little attention has been paid to their needs [1]. Informal caregivers contribute to the health system and constitute an important shadow workforce [2]. For example, in the USA, each year, almost a quarter of the population provides unpaid assistance to ill, disabled, or elderly persons [3].

Caregivers have to balance their caring responsibilities with employment and financial security, their own physical and mental health, and overall quality of life [4]. It is vital to understand the emotional stress and psychological health experienced by caregivers [5]. Caregivers who face social and emotional burdens related to caregiving have more health-risk behaviors such as alcohol use [6]. A meta-analysis study on health correlates of caregiving found that predictors of physical health are different from psychological health hence both merit theoretical and empirical attention [7]. Chronic physical conditions among caregivers tend to be worsen over time [8]. One such condition is lower back pain [9,10].

Information on the prevalence, health and wellbeing of caregivers in middle-income economies is quite limited. Some studies on caregivers have been reported from Asia but most of these have been conducted in more affluent countries. For example, there are reports from Japan on stress and female family caregivers [11-13]. A study in Hong Kong noted adverse physical and psychological health, and poor quality of life among caregivers of the elderly [14]. A report from Korea emphasized the role of the mental and the physical health of caregivers in predicting their overall health, regardless of impairment of the care recipients [15].

There is an urgent need for studies of caregiving in countries with emerging economies. Thailand is such a country and its caregiving needs are substantial. According to the Thai National Statistical Office’s 2007 Disability Survey [16], 1.8 million Thais are disabled and the majority requires caregivers [17]. Thai culture has characteristics that could influence caregiving and its consequences. For example, a strong Buddhist culture affects norms and values in caring for family members and would be expected to make unpaid caregiving quite common.

Thailand is facing one of the world’s most rapid processes of population ageing [18]. In 2000, 17% of Japanese, 16% of British, 13% of Australian, and 6% of Thai people were aged over 65 years. By 2025 these estimates are projected to rise to 30%, 19%, 19% and 13% respectively, with the highest proportionate increase (more than double) being for Thailand [18]. The Thai projection reflects better health care for the elderly and has as a consequence an increase in the need for caregivers [19]. According to the Thai National Statistical Office’s 2007 Survey of Thai Elderly [20], 10% of Thai elderly require caregivers (40% received care from daughters, 28% from spouses and 12% from sons). When asked ‘what type of welfare government should provide’ over 95% of elderly Thais preferred combined daycare centres and home care for elderly with chronic illnesses rather than formal institutional care. As Thais live longer, caregivers will become even more important to healthcare systems.

Our study focuses on family caregivers among adult working Thais residing throughout the country. Our objective is to investigate both physical and mental health among Thai adult caregivers and compare these to non-caregivers. Findings will be useful for health and social service providers and policymakers. Our results will facilitate identification of vulnerable caregivers to provide support and necessary resources.

## Methods

### Data

The Thai Health-Risk Transition project was established in 2005 by Thai investigators from Sukhothai Thammathirat Open University (STOU), the National Economic and Social Development Board, the Ministry of Public Health and Chiang Mai University, with Australian collaborators from the Australian National University and University of Queensland.

The aim of the project was to study the health-risk transition within the Thai population as it moved away from traditional patterns of illness (maternal and child mortality and infectious diseases) to emerging chronic diseases and injury. Distance-learning Thai adults living and working all over Thailand and enrolled at STOU were recruited. This cohort of individuals represents well the adult Thai population for median income, geographical location, and religion. They are generally persons of modest means with work and family commitments. Participation in the study was voluntary and study leaders reassured participants that their personal responses were confidential. Participants were motivated by being fully informed about the purposes of the Thai Health-Risk Transition study and that they could contribute to public health knowledge in Thailand. A periodic newsletter provides information back to participants on study progress. Details on enrolment and overall methodology have been reported elsewhere [21]. In 2005, the 20-page baseline questionnaires were mailed out to approximately 200,000 STOU students; the response rate was 44% and respondents were aged between 15 to 87 years (n=87,134). The questionnaire covered a wide range of topics including demographic, socioeconomic and geographic characteristics, health status, health service use, risk behaviours, injuries, dietary intake, and family background. The second questionnaire was sent to follow-up on participants in 2009 (n = 60,569, response rate 69.5%) with questions on socio-demographic characteristics, health and wellbeing, injury, health-risk behaviours, and disease [22]. The 2009 data also included a question regarding caregiver status which was not available in 2005; accordingly, this study is confined to the 2009 data.

### Measures and definitions

Caregiver in this study refers to a person who provides unpaid care to family members with disability, mental illness, or who are of frail or aged. Caregiver status was ascertained by the question: “Do you regularly care for a sick or disabled family member (‘No’, ‘Yes, part-time’ and ‘Yes, full-time’)?”

Measures of health were assessed as follows:

Self-assessed health was the first question of the Medical Outcomes Short Form instrument (SF8) which asked “Overall, how would rate your health during the past 4 weeks?” Answers were ‘excellent’, ‘very good’, ‘good’, ‘fair’, ‘poor’, and ‘very poor’. For analysis we dichotomised the responses, combining the ‘poor’ and ‘very poor’ groups as having ‘poor self-rated health’.

Psychological distress was based on the standard Kessler 6 instrument which included questions “In the past 4 weeks, how often did you feel: 1) so sad nothing could cheer you up, 2) nervous, 3) restless or fidgety, 4) hopeless, 5) everything was an effort, 6) worthless?” Responses were scored from 0 ‘none of the time’, 1 ‘a little of the time’, 2 ‘some of the time’, 3 ‘most of the time’, 4 ‘all the time’ [23,24]. Scores for the 6 questions were then combined; those with a total score ≥ 13 (out of the possible 24) were classified as having ‘psychological distress’.

Lower back pain was assessed using two ‘yes-no’ questions: “In the past 4 weeks, have you had pain in your lower back?” and “If yes, was this pain bad enough to limit your usual activities or change your daily routine for more than one day”? These questions have been standardised by back pain specialists [25]. For our analysis, we classified those who answered ‘yes’ to both questions as having ‘lower back pain’.

Health covariates include:

Body Mass Index (BMI) was calculated from self-reported height and weight and was then categorised in accordance with the International Obesity Task Force cut-offs for Asian populations: BMI≤18.5 ‘underweight’, 18.5<BMI<23 ‘normal’, 23<BMI<25 ‘overweight at risk’, and BMI≥25 ‘obese’ [26].

Health behaviours such as smoking (≥ 3 cigarettes per day) and drinking (>10 glasses per week or ≥ 5 days per week) were also recorded.

Analyses were stratified by sex as various studies have found differences in health and needs among male and female caregivers [11,27,28]. As well as caregiver status, characteristics of study participants examined in this report were sex, age, marital status, household monthly income, work status, and geographical residence.

### Data analyses

Data scanning and editing were conducted using Thai Scandevet software. Further data editing of the baseline study was completed using SQL and SPSS software. Individuals with missing data for given analyses were excluded so totals varied according to the information available. Participants with unknown caregiving status (n=874) were excluded from the multivariate analyses. For analysis we used Stata version 12 reporting crude odds ratios from bivariate logistic regression and adjusted odds ratios from multivariate logistic regression. We have included potential confounders of health measures based on previous published papers on adverse health among the study participants [29-32]. In addition to this previous experience with explanatory variables, our approach to identifying confounders was reinforced with bivariate analyses which show strong association between explanatory variables and health measures. There were some exceptions in exposure-health associations; for example age was not strongly associated with lower back pain among males. However, in view of the overall biological influence of age, we include age groups as explanatory variables in all of our analyses.

Correlations between explanatory variables were also tested and the highest relationship was found between age and income (r = 0.40); the rest of the correlation coefficients were less than 0.30. We then used Variance Inflation Factor (VIF) regression statistics to test for multi-collinearity in all models; VIFs ranged from 1.08 to 4.14 and as these values were less than 10 there was no evidence of multi-collinearity. Accordingly, all explanatory variables were retained in the final model.

### Ethical issues

Ethics approval was obtained from Sukhothai Thammathirat Open University Research and Development Institute (protocol 0522/10) and the Australian National University Human Research Ethics Committee (protocol 2004/344 and 2009/570). Informed written consent was obtained from all participants.

## Results

Among study participants in 2009, 27.5% reported being part-time and 6.6% reported being full-time caregivers (Table 1). Approximately equal proportions of males and females were caregivers. Caregiving was more common in the older age group—13.9% of non-caregivers were aged 45 and above compared to 18.2% of part-time caregivers and 23.0% of full-time caregivers. Full-time caregivers, compared to non-caregivers, were more likely to be married (65.6% vs 54.0%), being unpaid family members (11.4% vs 6.6%), and to be rural residents (51.7% vs 41.4%). Caregivers were more likely to engage in health risk behaviours including smoking and drinking and reported higher body mass index compared to non caregivers. Also tabulated were participants who did not report their caregiver status; they were more likely to be males, in older age groups, with lower income, less paid work, and more frequent smoking and drinking. It is noteworthy that these differences were quite small.

**Table 1:** Characteristics of participants by caregiver status 2009

**Participant characteristics**	**Participants% (n=60569)**	**Caregiver status**			
		**Non-caregiver**	**Part-time caregiver**	**Full-time caregiver**	**Unknown status**
		**(n=39350)**	**(n=16436)**	**(n=3909)**	**(n=874)**
**Demographic characteristics**					
Sex					
Male	45.3 (27407)	43.8	47.5	48.4	54.3
Female	54.8 (33162)	56.2	52.5	51.6	45.8
Age (year)					
20-29	27.4 (16591)	29.7	24.5	18.0	21.4
30-44	56.9 (34455)	56.5	57.4	59.0	57.6
45+	15.7 (9523)	13.9	18.2	23.0	21.0
Marital status					
Married	55.3 (30490)	54.0	55.8	65.6	55.9
Never married	37.9 (20927)	39.3	34.5	26.1	36.0
Separated, divorced, widowed	6.8 (3768)	6.7	6.7	8.3	8.2
**Socio-geographic characteristics**					
Household monthly income (Baht)*					
<10,000	18.8 (11004)	17.9	20.2	20.8	24.7
10,000-19,999	22.4 (13129)	23.4	21.0	18.9	24.0
20,000-30,000	35.7 (20891)	36.3	35.1	33.3	31.6
>30,000	23.1 (13513)	22.5	23.8	27.0	19.7
Work status					
Doing paid work	73.2 (44332)	74.9	71.1	66.5	63.4
Unpaid family workers	7.3 (4405)	6.6	7.9	11.4	6.9
Seeking work	2.2 (1334)	2.1	2.3	2.6	2.3
Others	17.3 (10498)	16.3	18.7	19.5	27.5
Geographical residence					
Rural residence	44.0 (26052)	41.4	48.2	51.7	48.2
Urban residence	56.0 (33144)	58.6	51.9	48.3	51.8
**Health covariates**					
Regular smokers - yes	7.7 (4659)	7.3	8.4	8.0	10.3
Regular alcohol drinkers - yes	13.7 (8269)	13.0	14.2	15.5	23.5
Body Mass Index					
Underweight	9.5 (5645)	10.1	8.8	6.9	8.4
Normal	49.5 (29406)	50.0	49.0	47.9	46.3
Overweight at risk	18.8 (11159)	18.3	19.6	19.1	23.4
Obese	22.1 (13148)	21.6	22.6	26.1	21.9

*1 $US ~ 35 Thai Baht.

Measures of health are analysed for study participants overall and also separately for males and females by caregiver status (Table 2). Poor self-assessed health was more commonly reported among females than males (6.0%, 7.3%, 7.8% compared to 4.1%, 4.7%, 4.6% among non-, part-, and full-time caregivers). Crude odds ratios for caregiver status and self-assessed health from bivariate logistic regression were only significant among females. Lower back pain and psychological distress were strongly associated with caregiver status. Those with unknown caregiver status (n=874) have associations with health but there was no systematic tendency to be similar to any particular group whose caregiver status was reported; this implies the group of 874 includes combination of participants from the three caregiving categories.

**Table 2:** Physical and mental health outcomes by sex and caregiver status 2009

**Health outcomes by sex**	**Caregiver status**			
	**Non-caregiver (n=39350)**	**Part-time caregiver (n=16436)**	**Full-time caregiver (n=3909)**	**Unknown status (n=874)**
**Male**				
Self-assessed health: poor or very poor	4.1 (704)*	4.7 (364)	4.6 (87)	4.0 (16)
Crude Odds Ratios [95% CI]	1.00**	1.15 [1.01-1.31]	1.13 [0.90-1.42]	0.98 [0.59-1.62]
Lower back pain: limit daily activity >1 day	3.9 (648)	5.3 (416)	7.2 (127)	6.8 (15)
Crude Odds Ratios [95% CI]	1.00	**1.47** [1.29-1.67]	**1.89** [1.55-2.30]	**1.79** [1.05-3.04]
Psychological distress: Kessler 6 score ≥ 13	4.1 (703)	4.7 (365)	6.1 (113)	4.7 (20)
Crude Odds Ratios [95% CI]	1.00	**1.15** [1.02-1.31]	**1.49** [1.22-1.83]	1.14 [0.72-1.80]
**Female**				
Self-assessed health: poor or very poor	6.0 (1332)	7.3 (627)	7.8 (157)	6.3 (21)
Crude Odds Ratios [95% CI]	1.00	**1.22** [1.11-1.35]	**1.32** [1.11-1.56]	1.04 [0.66-1.62]
Lower back pain: limit daily activity >1 day	3.4 (749)	5.3 (444)	6.9 (135)	4.5 (9)
Crude Odds Ratios [95% CI]	1.00	**1.55** [1.38-1.75]	**2.06** [1.71-2.49]	1.31 [0.67-2.57]
Psychological distress: Kessler 6 score ≥ 13	5.6 (1221)	7.2 (617)	8.4 (166)	7.0 (26)
Crude Odds Ratios [95% CI]	1.00	**1.32** [1.19-1.46]	**1.53** [1.30-1.82]	1.26 (0.84-1.89)

*% prevalence (frequency) of each health outcome.

**Odds Ratios relating health outcomes to caregiver status (non caregiver as reference).

Results from multivariate logistic regressions are tabulated separately for males and females (Tables 3 and 4). After accounting for possible socio-demographic and health covariates, compared to male non-caregiver counterparts, being a part- or full-time male caregiver was associated with lower back pain (Adjusted Odds Ratios 1.36 and 1.67) and psychological distress (AOR = 1.16 and 1.68). Caregiver status was not significantly associated with ‘poor or very poor’ self-assessed health for males (Table 3). Compared to female non-caregivers (Table 4), being a part- or full-time female caregiver had effects on health: ‘poor or very poor’ self-assessed health (AOR = 1.21 and 1.34), lower back pain (AOR = 1.47 and 1.84) and psychological distress (AOR = 1.32 and 1.52).

**Table 3:** Health outcomes* for males related to caregiver status 2009 (adjusted Odds Ratios and 95% Confidence Intervals)**

**Participant characteristics**	**Poor self-assessed health**	**Lower back pain**	**Psychological distress**
**Males (n=27407)**	(n=23519)**	**(n=23233)**	**(n=22781)**
**Caregiver status**			
Non caregiver	1.00	1.00	1.00
Part-time caregiver	1.15 [0.90-1.32]	**1.36** [1.19-1.57]	**1.16** [1.01-1.34]
Full-time caregiver	1.14 [0.89-1.47]	**1.67** [1.34-2.08]	**1.68** [1.32-2.09]
**Demographic characteristics**			
Age (year)			
20-29	**1.50** [1.17-1.93]	0.91 [0.72-1.16]	**1.44** [1.12-1.84]
30-44	**1.48** [1.22-1.79]	0.99 [0.83-1.18]	**1.29** [1.05-1.59]
45+	1.00	1.00	1.00
Marital status			
Married	1.00	1.00	1.00
Never married	1.16 [0.99-1.35]	**0.72** [0.60-0.85]	**1.33** [1.13-1.55]
Separated, divorced, widowed	**1.37** [1.05-1.78]	1.13 [0.87-1.47]	**2.02** [1.58-2.58]
**Socio-geographic characteristics**			
Household monthly income (Baht)			
<10,000	1.10 [0.87-1.39]	**2.09** [1.66-2.62]	**2.38** [1.88-3.02]
10,000-19,999	1.01 [0.82-1.25]	**1.64** [1.32-2.03]	**1.80** [1.43-2.26]
20,000-30,000	0.93 [0.79-1.11]	**1.21** [1.01-1.46]	**1.27** [1.04-1.56]
>30,000	1.00	1.00	1.00
Work status			
Doing paid work	1.00	1.00	1.00
Unpaid family workers	1.23 [0.93-1.63]	**1.49** [1.16-1.92]	1.19 [0.90-1.57]
Seeking work	1.33 [0.87-2.02]	**1.82** [1.26-1.62]	**2.94** [2.19-3.94]
Others	1.08 [0.91-1.29]	**1.19** [1.01-1.42]	1.17 [0.99-1.39]
Geographical residence			
Rural residence	1.00	1.00	1.00
Urban residence	**1.15** [1.01-1.32]	0.89 [0.78-1.01]	**1.21** [1.06-1.38]
**Health-risk behaviours**			
Regular smokers - no	1.00	1.00	1.00
Regular smokers - yes	**1.53** [1.31-1.78]	**1.31** [1.11-1.53]	**1.41** [1.20-1.65]
Regular alcohol drinkers - no	1.00	1.00	1.00
Regular alcohol drinkers - yes	**1.18** [1.02-1.37]	**1.15** [0.90-1.25]	**1.35** [1.17-1.57]
Body Mass Index			
Underweight	**1.67** [1.24-2.26]	**1.23** [0.89-1.71]	**1.35** [1.02-1.79]
Normal	1.00	1.00	1.00
Overweight at risk	1.13 [0.95-1.34]	1.06 [0.90-1.25]	0.91 [0.77-1.08]
Obese	**1.62** [1.39-1.89]	**1.21** [1.03-1.41]	**1.11** [0.95-1.30]

*self-assessed health: ‘poor or very poor’; lower back pain: limits daily activity > 1 day; Kessler 6: score ≥ 13.

**multivariate analyses (numbers varied for each outcome): logistic regression included all attributes listed in Table.

**Table 4:** Health outcomes* for females related to caregiver status 2009 (adjusted Odds Ratios and 95% Confidence Intervals)**

**Participant characteristics**	**Poor self-assessed health**	**Lower back pain**	**Psychological distress**
**Females (n=33162)**	(n=28266)**	**(n=28000)**	**(n=27651)**
**Caregiver status**			
Non caregiver	1.00	1.00	1.00
Part-time caregiver	**1.21** [1.10-1.35]	**1.47** [1.30-1.70]	**1.32** [1.18-1.48]
Full-time caregiver	**1.34** [1.11-1.61]	**1.84** [1.49-2.27]	**1.52** [1.25-1.83]
**Demographic characteristics**			
Age (year)			
20-29	1.07 [0.88-1.30]	0.97 [0.77-1.23]	**1.81** [1.44-2.27]
30-44	**1.24** [1.05-1.47]	0.93 [0.76-1.14]	**1.40** [1.13-1.73]
45+	1.00	1.00	1.00
Marital status			
Married	1.00	1.00	1.00
Never married	1.06 [0.96-1.18]	**0.78** [0.68-0.90]	**1.21** [1.09-1.36]
Separated, divorced, widowed	**1.38** [1.17-1.63]	1.05 [0.85-1.31]	**1.97** [1.67-2.33]
**Socio-geographic characteristics**			
Household monthly income (Baht)			
<10,000	**1.26** [1.06-1.50]	**1.76** [1.42-1.20]	**2.16** [1.77-2.63]
10,000-19,999	**1.18** [1.01-1.38]	**1.31** [1.06-1.62]	**1.78** [1.47-2.14]
20,000-30,000	1.15 [1.00-1.33]	**1.14** [0.93-1.39]	**1.54** [1.29-1.84]
>30,000	1.00	1.00	1.00
Work status			
Doing paid work	1.00	1.00	1.00
Unpaid family workers	0.94 [0.78-1.12]	**1.26** [1.02-1.54]	1.05 [0.88-1.26]
Seeking work	**1.43** [1.04-1.96]	**1.58** [1.10-2.26]	**2.36** [1.83-3.06]
Others	**1.16** [1.01-1.33]	**1.48** [1.26-1.74]	**1.20** [1.05-1.38]
Geographical residence			
Rural residence	1.00	1.00	1.00
Urban residence	**1.24** [1.12-1.31]	1.05 [0.93-1.19]	**1.13** [1.02-1.25]
**Health-risk behaviours**			
Regular smokers - no	1.00	1.00	1.00
Regular smokers - yes	**2.26** [1.43-3.55]	1.77 [0.95-3.32]	**1.29** [1.44-3.65]
Regular alcohol drinkers - no	1.00	1.00	1.00
Regular alcohol drinkers - yes	**1.12** [0.93-1.35]	**1.37**[1.10-1.70]	**1.20** [1.01-1.46]
Body Mass Index			
Underweight	1.02 [0.88-1.19]	0.90 [0.88-1.19]	1.03 [0.89-1.20]
Normal	1.00	1.00	1.00
Overweight at risk	**1.27** [1.10-1.46]	**1.28** [1.07-1.52]	**1.24** [1.07-1.44]
Obese	**1.54** [1.36-1.74]	**1.58** [1.35-1.85]	**1.21** [1.05-1.39]

*self-assessed health: ‘poor or very poor’; lower back pain: limits daily activity > 1 day; Kessler 6: score ≥ 13.

**multivariate analyses (numbers varied for each outcome): logistic regression included all attributes listed in Table.

Younger participants were more likely to report psychological distress than older participants; this was also noted for those who were never married, divorced, separated or widowed compared to married respondents. Currently seeking work, smoking and drinking were also associated with psychological distress. Belonging to the lowest income group, smoking and obesity were all associated with ‘poor or very poor’ self-assessed health and lower back pain. These associations indicate the need to include these covariates in the analyses of caregiver status and health.

## Discussion

We examined the physical and mental health of adult Thai caregivers. Taking into account possible confounders, being a caregiver was associated with lower back pain and psychological distress among males and females and ‘poor or very poor’ self-assessed health only among females. This could partly be explained by gender difference in reporting self-assessed health among middle-aged adults. Previously we found that females were much more likely to perceive and report worse self-assessed health than males [30]. We conducted a telephone follow-up of 120 caregivers and found that one-third has been caring for 1–2 years and two-thirds have been caring for 3–5 years. Over 60% reported cause of caring was illness. Assistance included mobility, provision of food and medication, as well as help with daily care.

Our study supports other reports on caregivers and their health, but unlike most work on this topic, our results derive from a developing economy in Asia. Generally, the effects of caring in Thailand are quite similar to those reported in rich, developed countries. For example, in the USA, higher levels of stressors among caregivers have been associated with poor self-reported health, more negative health behaviors, and greater use of health care services [33]. One study in the UK also found caregiving at home to be associated with morbidity, bodily pain, and obesity [34]. However, one of the main differences in lower income nations was the limited formal social welfare support system for family caregivers. This could further exacerbate the caregiver burden in emerging economies.

Depression experienced by the caregivers may negatively impact the care recipient, which may further limit self-care and functioning abilities, thus necessitating additional assistance [35]. Emphasis should also be placed on interventions during the transition to and adjustment into caring roles [36]. A prospective, British population-based study highlighted that transition into and out of unpaid caregiving is associated with increased risk for onset of or delayed recovery from psychological distress [36]. One report emphasised the importance of effective caregiver support and early health promotion for care recipients, monitoring high risk groups, and timing interventions [37]. Exercise programs for caregivers could also help if focused on preventing back pain by developing endurance strength [38].

There is a need for a coordinated system that makes easier the complex work of family caregivers by providing the training and support needed. In order for caregivers to maintain their wellbeing, various studies highlight the need for information in areas including finance, law, and health [39-41]. A qualitative study of informal caregiving provided to elderly stroke survivors in Thailand highlighted caregiver needs for information, assistance, and support [42]. Relevant Thai studies on family caregivers reported social support to be vitally important for both caregivers and care recipients among impaired Thai older adults [43,44].

Cultural differences among caregivers should be taken into account. For example, differences were found in level of stress and coping mechanisms among Korean, Korean-American and Caucasian-American caregivers [45]. Caucasians reported affection while Koreans and Korean Americans reported filial obligation as their motivation for caregiving. In addition, Korean caregivers reported higher extended family support than Caucasian caregivers, while Caucasian caregivers reported higher utilisation of formal support than Korean caregivers. In Thailand, Buddhist concepts are viewed by many as part of daily life, for example, the return of good karma by caring for the loved ones in the family. Our earlier study has found Thai adults strongly affirming their belief in karma and the importance of religion to calm one’s mind [46].

As noted in the results of this study, 874 participants did not report their caregiving status and were excluded from analyses. Could this relatively small group bias the results? Setting the values for the 874 non-responding participants according to three scenarios (1. ‘all non-caregivers’; 2. ‘all part-time caregivers’; 3. ‘all full-time caregivers’) enables new estimates on health associations (e.g., lower back pain). These estimates differ little from the tabulated estimates shown in Tables 3 and 4. For example, without the 874 non-responding participants, the back pain estimates for males were AORs 1.36 and 1.67; with the 874 non-responding participants set to ‘all non-caregivers’ AORs became 1.38 and 1.71, set to ‘all part-time caregivers’ AORs became 1.38 and 1.72, set to ‘full-time caregivers’ AORs became 1.39 and 1.66. We conclude that the bias was minimal and did not change the epidemiological results.

The strength of this study is its large national scale with its wide array of socio-demographic, health-risk behaviours and measures of health available. Caution should be applied when interpreting the findings: our study is based on a group of long-distance adult students aspiring to improve their modest socioeconomic circumstances. The causes of adverse physical and mental health in our study may be different than those among caregivers in the general population. Further in-depth study on the nature and type of caregiving among Thai adults will provide insights into the long term social and health outcomes of caregiving and the support they require [12,47].

## Conclusion

Our study found that caregiving among Thai adults was strongly associated with adverse health. Further, these findings were consistent across physical and mental health. Our findings should raise awareness of the unmet needs of caregivers, and the need for support of caregivers in Thailand and other similar middle-income countries.

## Abbreviations

AOR: Adjusted Odds Ratios; CI: Confidence Interval; VIF: Variance Inflation Factor.

## Competing interests

The authors that they have no competing interests.

## Authors’ contributions

VY conceptualised, analysed, and drafted the manuscript. DH provided expert advice on disability and caregivers. SS and AS devised and directed the Thai Health-Risk Transition project. AS provided editorial guidance on the revisions. All authors approved the final manuscript submission.

## Thai cohort study team

Thailand: Jaruwan Chokhanapitak, Suttanit Hounthasarn, Suwanee Khamman, Daoruang Pandee, Suttinan Pangsap, Tippawan Prapamontol, Janya Puengson, Sam-ang Seubsman, Boonchai Somboonsook, Nintita Sripaiboonkij, Pathumvadee Somsamai, Duangkae Vilainerun, Wanee Wimonwattanaphan, Cha-aim Pachanee, Wimalin Rimpeekool, Tewarit Somkotra, Arunrat Tangmunkongvorakul, Benjawan Tawatsupa.

Australia: Chris Bain, Emily Banks, Cathy Banwell, Bruce Caldwell, Gordon Carmichael, Tarie Dellora, Jane Dixon, Sharon Friel, David Harley, Matthew Kelly, Tord Kjellstrom, Lynette Lim, Anthony McMichael, Tanya Mark, Adrian Sleigh, Lyndall Strazdins, Susan Jordan, Janneke Berecki-Gisolf, Roderick McClure, Vasoontara Yiengprugsawan.

## Pre-publication history

The pre-publication history for this paper can be accessed here:

http://www.biomedcentral.com/1471-2458/12/1111/prepub
